# The near complete mitochondrial genome of *Oreolalax schmidti* (Anura: Megophryidae)

**DOI:** 10.1080/23802359.2020.1806134

**Published:** 2020-10-09

**Authors:** Xingli Bao, Lin Cui, Diyan Li, Xiaolan Fan, Mingyao Yang, Deying Yang, Qingyong Ni, Yongfang Yao, Huailiang Xu, Bo Zeng, Feida Sun, Mingwang Zhang

**Affiliations:** a College of Animal Science and Technology, Sichuan Agricultural University, Chengdu, PR China; b Animal Genetic Resources Exploration and Innovation Key Laboratory of Sichuan Province, Sichuan Agricultural University, Chengdu, Sichuan, China; c College of Life Science, Sichuan Agricultural University, Ya’an, Sichuan, PR China

**Keywords:** Near mitochondrial genome, *Oreolalax schmidti*, phylogenetic analysis

## Abstract

We newly obtained the near complete mitochondrial genome of *Oreolalax schmidti* (18,481bp) by using polymerase chain reaction (PCR) in this study. It includes 13 protein-coding genes (PCG), two ribosomal RNA (rRNA) genes, and 23 transfer RNA (tRNA) genes. The phylogenetic tree indicates that the *Oreolalax schmidti* is closely related to the *O. lichuanensis*. This report will help the further studies of *Oreolalax* species classification and source protection.

The *Oreolalax schmidti* belongs to the family Megophryidae, genus *Oreolalax,* which is endemic to China (Fei et al. 2012; Frost 2020) and distributed in Central Sichuan (Baoxing, Dujiangyan, Mount Emei, Hongya, Mianning, Shimian, and Wenchuan counties). This species lives in the mountain, regions at an altitude of 1700-2400 m, residing in bushes near small streams, in damp caves or under rocks in the streams, currently suffering from habitat loss (AmphibiaChina 2020). However, there is only a small portion of its mitochondrial genomes of *O. schmidti* available in the GenBank, e.g., 12 s rRNA and 16 s rRNA genes. This study along with the first near complete mitochondrial genome of *O. schmidti* may help us understand the information and phylogeny of this species.

During the field trip in 2015, the sample of *O. schmidti* was collected from Wawushan Nature Reserve (29°30′28.32″N, 102°51′53.20″E., elev. 2071 m), Sichuan Province, China, and the tissue was preserved in 99.9% ethanol. The specimen was deposited at the Museum of Sichuan Agricultural University (Specimen voucher: 20120281). Genomic DNA was extracted from leg muscle tissue by using the Ezup pillar genomic DNA extraction kit (Sangon Biotech, Shanghai, China). Finally, we sent DNA sample to Personal Biotechnology (Shanghai, China) for library construction and sequencing on an Illumina MiSeq platform (PE400).

The near complete mitogenome (MT773151) of *O. schmidti* is 18,481bp in length, which contained 38 genes including 13 protein coding genes (PCGs), two ribosomal RNA (rRNA) genes, 23 tRNA genes (including two *trnM* genes) and a partial control region sequence (about 1,498bp). The majority mitogenome length of *O. schmidti* is 18,481, and the base composition of the mitogenome is 28.3% A, 14.4% G, 24.5% C and 32.8% T, respectively. Comparative analysis indicates that the mitogenome structure of *O. schmidti* is similar to that of other Megophryidae (Xiang 2013; Liang 2016; Liang et al. 2016; Huang et al. 2020). *Nad6* and eight *tRNA* (*tRNA-Gln, Ala, Asn, Cys, Tyr, Ser, Glu, and Pro*) are located on the L-light strand (L strand), and the others are encoded on the heavy strand (H strand). Most of the PCG_S_ (nine) were started by ATG, except three PCG_S_ (*cox1, nad5, and nad6*) by GTG, and *nad3* by ATT. *Cox1* was terminated by TAG, two PCG_S_ (*nad5 and nad6*) terminated by AGG as termination codon, and four (*nad2, atp8, nad3 and nad4l*) terminated by TAA. Four PCG_S_ (*nad1, atp6, cox3 and nad4*) stopped with TA, whereas two (*cox2 and cytb*) stopped with a single base T. The length of 12S and 16S rRNA are 938 and 1,581 bp respectively.

In order to determine the taxonomic status of *O. schmidti*, we constructed the phylogenetic tree by MEGA7.0 (Kumar et al. 2016) based on 13 PCGs. The final alignment consisted of 23 sequences of 20 species from eight genera (*Oreolalax, Leptobrachium, Scutiger, Leptolalax, Atympanophrys, Megophrys, Xenophrys*, and *Xenopus*), and we select three *Xenopus* species as outgroups. The phylogenetic trees indicated *O. schmidti* has the closest relationship with *O. lichuanensis*, and a monophyletic clade of genus *Oreolalax* were recovered and well sported with 100 percent bootstrap support (Figure 1). This near complete mitogenome would contribute to further investigations of molecular evolution and conservation of genus *Oreolalax*.

**Figure 1.**
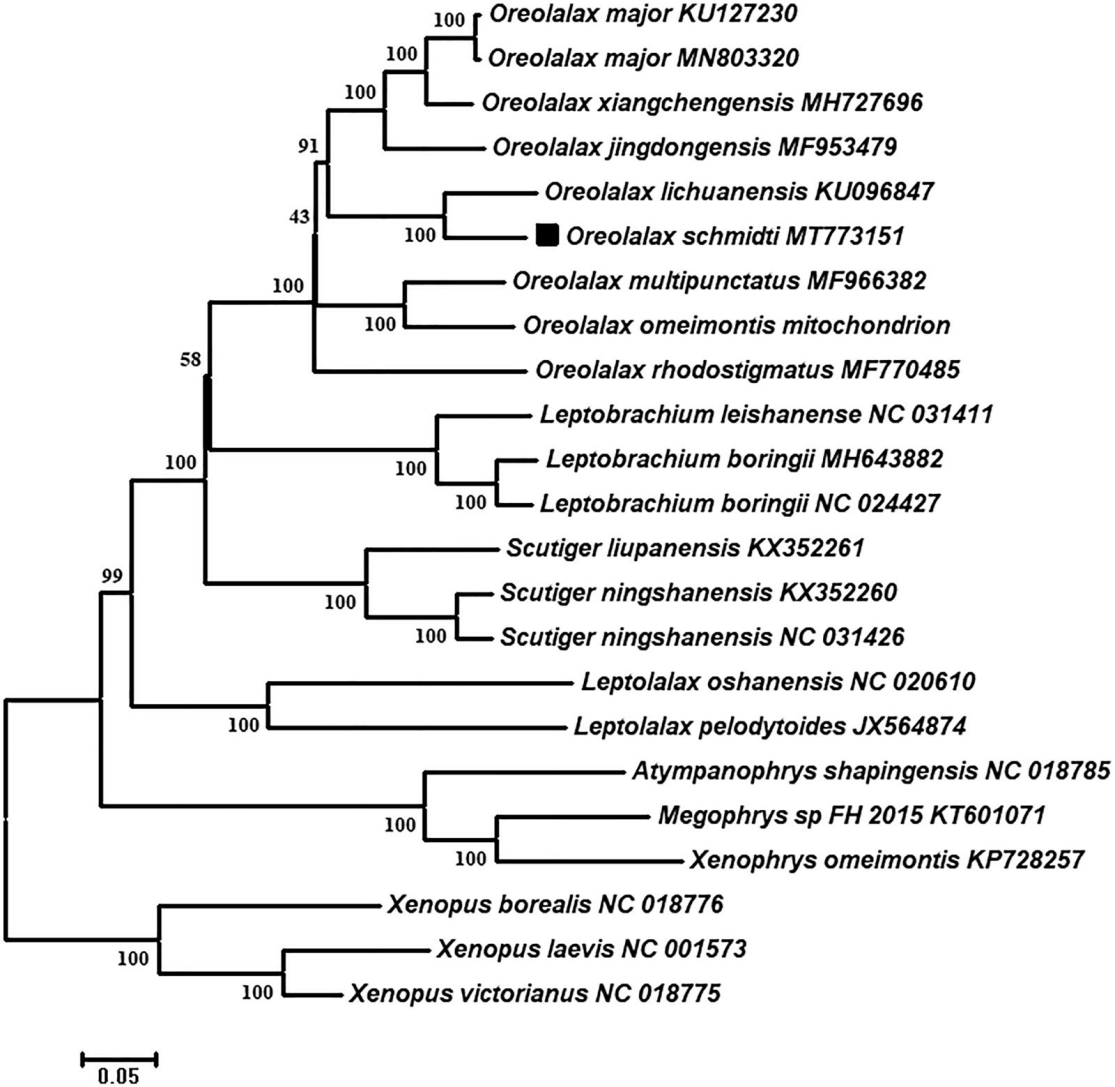
Neighbour-joining (NJ) phylogenetic tree based on all 13 combined mitochondrial protein-coding genes from 20 species. The numbers of internal branches are bootstrap values.

## Data Availability

The data used to support the findings of this study are available in GenBank at https://www.ncbi.nlm.nih.gov/nuccore/ MT773151, reference number MT773151.
